# Determinants of Indices of Cerebral Volume in Former Very Premature Infants at Term Equivalent Age

**DOI:** 10.1371/journal.pone.0170797

**Published:** 2017-01-26

**Authors:** Aurelie Naud, Emmanuelle Schmitt, Maelle Wirth, Jean-Michel Hascoet

**Affiliations:** 1 Department of Neonatology, Maternité Régionale, CHRU NANCY, France; 2 Department of Neuroradiology, CHRU NANCY, France; 3 EA 3450 - DevAH, Université de Lorraine, Nancy, France; Centre Hospitalier Universitaire Vaudois, FRANCE

## Abstract

Conventional magnetic resonance imaging (MRI) at term equivalent age (TEA) is suggested to be a reliable tool to predict the outcome of very premature infants. The objective of this study was to determine simple reproducible MRI indices, in premature infants and to analyze their neonatal determinants at TEA. A cohort of infants born before 32 weeks gestational age (GA) underwent a MRI at TEA in our center. Two axial images (T2 weighted), were chosen to realize nine measures. We defined 4 linear indices (MAfhlv: thickness of lateral ventricle; CSI: cortex-skull index; VCI: ventricular-cortex index; BOI: bi occipital index) and 1 surface index (VS.A: volume slice area). Perinatal data were recorded. Sixty-nine infants had a GA (median (interquartile range)) of 30.0 weeks GA (27.0; 30.0) and a birth weight of 1240 grams (986; 1477). MRI was done at 41.0 (40.0; 42.0) weeks post menstrual age (PMA). The inter-investigator reproducibility was good. Twenty one MRI (30.5%) were quoted abnormal. We observed an association with retinopathy of prematurity (OR [95CI] = 4.205 [1.231–14.368]; p = 0.017), surgery for patent ductus arteriosus (OR = 4.688 [1.01–21.89]; p = 0.036), early onset infection (OR = 4.688 [1.004–21.889]; p = 0.036) and neonatal treatment by cefotaxime (OR = 3.222 [1.093–9.497]; p = 0.03). There was a difference for VCI between normal and abnormal MRI (0.412 (0.388; 0.429) vs. 0.432 (0.418; 0.449); p = 0,019); BOI was higher when fossa posterior lesions were observed; VS.A seems to be the best surrogate for cerebral volume, 80% of VS.As’ variance being explained by a multiple linear regression model including 7 variables (head circumference at birth and at TEA, PMA, dopamine, ibuprofen treatment, blood and platelets transfusions). These indices, easily and rapidly achievable, seem to be useful but need to be validated in a large population to allow generalization for diagnosis and follow-up of former premature infants.

## Introduction

Despite decreasing incidence, cerebral lesions remain an important issue for very premature infants’ neurodevelopment outcome [1, 2]. Early recognition of infants with cerebral lesions, at risk of delayed or abnormal neurodevelopment is important both for accurate counseling of parents and for selection of infants that may benefit from specific follow-up, allowing early behavioral interventions or rehabilitation services [3]. Conventional magnetic resonance imaging (MRI) at term equivalent age (TEA) has been suggested to be able to reliably predict the outcome of very premature infants [4]. An abnormal MRI in and of itself will lead to more vigilance in follow up, but a normal MRI is not always associated with a normal developmental outcome. Therefore, it is important to improve MRI specificity of former preterm infants with apparent “normal” MRI. Peterson et al. found that premature birth was associated with long term specific anatomical reductions in brain volume [5]. Other studies have shown a correlation between cerebral growth and alteration of neurodevelopment outcome in former premature infants [4–7]. Inder et al. demonstrated that premature infants with moderate to severe disability at one year of age have significant reduction in gray matter volume, both cortical and nuclei, and an increase in cerebrospinal fluid volume [6]. This correlation is stronger when white matter injury is associated [7].

Some of the factors influencing cerebral volume have been identified [8–10]. For instance, Murphy et al. found that early systemic postnatal treatment of premature infants by high dose of dexamethasone is associated with impaired brain growth. This was mainly seen at the level of cerebral cortical gray matter, without cerebral injury such as cystic periventricular leukomalacia (c-PVL) or intra ventricular hemorrhage (IVH). The total cerebral tissue volume was reduced by 30% in this population as compared to those who had not been treated [11]. Studies in rats, exposed to dexamethasone, have shown an increase of developmental apoptosis and an alteration of neuronal differentiation [12–14]. Children treated by dexamethasone had significantly poorer motor skills and coordination, lower full intelligence quotient (IQ) scores, verbal IQ scores, performance IQ scores and smaller head circumference [15–17].

The MRI study and analysis of the cerebral volume at TEA appear to be very useful [5, 7, 18]. However, volume segmentation is indeed difficult and time consuming and is not available in routine clinical setting. Geometrical, linear and surface indices might be a surrogate for brain volume to evaluate infants’ outcome. For instance, Ragan et al. demonstrated that the fronto-occipital horn ratio (FOHR) in hydrocephalus has a strong correlation with ventricular volume and this FOHR is in common use [19].

The objective of this study was to validate simple reproducible MRI geometrical and anatomical indices, in premature infants born before 32 gestational age (GA), and to analyze their neonatal determinants at TEA.

## Materials and Methods

### Subjects

In our Level III Center, all preterm infants born before 32 weeks GA undergo a routine MRI, performed at TEA, as part of their follow-up. All infants with their MRI scheduled between January 4^th^ and May 30^th^ 2016 were included in this study. Infants with a genetic syndrome or cerebral abnormality (brain malformations) were excluded. This prospective study was nested in an observational study (Etude Premavision, Wirth M, 2016) approved by our institutional research board (DRI, CHRU Nancy: PSS2016/PREMAVISION), registered to the Commission Nationale Informatique et Libertés (number: R2016-02) and ClinicalTrials.com (number: NCT02890251). Both parents were informed that the data will be used for research purposes and their non-opposition was obtained in written and signed.

### Data acquisition

MRI was performed when the infants were between 39^th^ and 41^st^ week post menstrual age (PMA). Prior to undergoing MRI, each infant was fed, wrapped in a blanket then placed, not sedated, in a 1.5 Tesla Vantage Titan^®^ of Toshiba tunnel, with cardiac frequency and oxygen saturation monitoring. For standard acquisition, different imaging modes were applied: T1-weighted (coronal, axial and sagittal), T2-weighted (axial and coronal), T2* weighted (axial) and diffusion (axial).

### MRI indices and analysis

**Fig 1:**
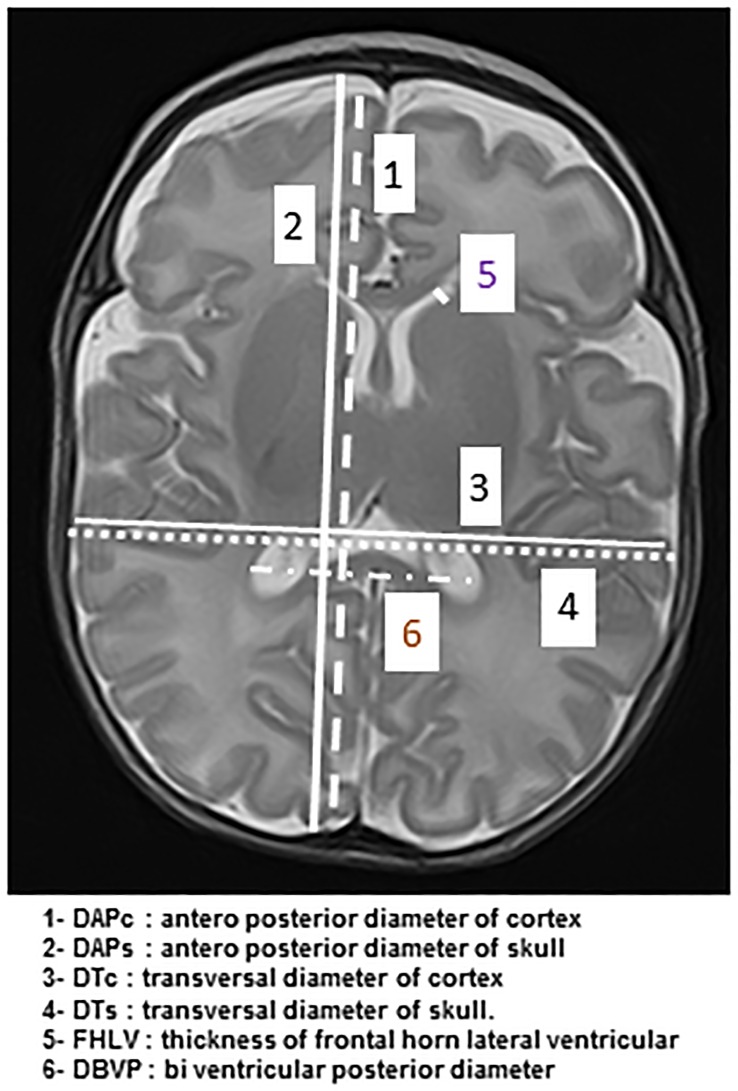
MRI linear indices on the axial image in T2-weighted including deep nuclei gray matter and Monro’s foramens. DAPc: anterior-posterior diameter of cortex /DAPs: anterior-posterior diameter of skull/ DTc: transversal diameter of cortex / DTs: transversal diameter of skull/ FHLV: thickness of frontal horn of lateral ventricle/ DBVP: biventricular posterior diameter.

Firstly, measures were done manually on the axial image in T2-weighted including deep nuclei gray matter and Monro’s foramens (3.3 mm axial slices; flip angle: 90°; repetition time: 7000; echo time: 100; matrix: 192 x 256). Images were acquired with a display field of view 14 cm x 18 cm. Two anterior-posterior fronto-parietal diameters were measured at the right hand side of the falx cerebri, to avoid the sagittal sinus, one for the cortex (DAPc) and one for the skull (DAPs), as well as two transversal diameters (tangent to the posterior edges of the thalamus) one for the cortex (DTc) and one for the skull (DTs). The thickness of the frontal horn of the left (FHLVL) and right (FHLVR) lateral ventricle was also measured. The biventricular posterior diameter (DBVP) was defined as the distance between the external edges of the ventricular crossroads (Fig 1).

**Fig 2:**
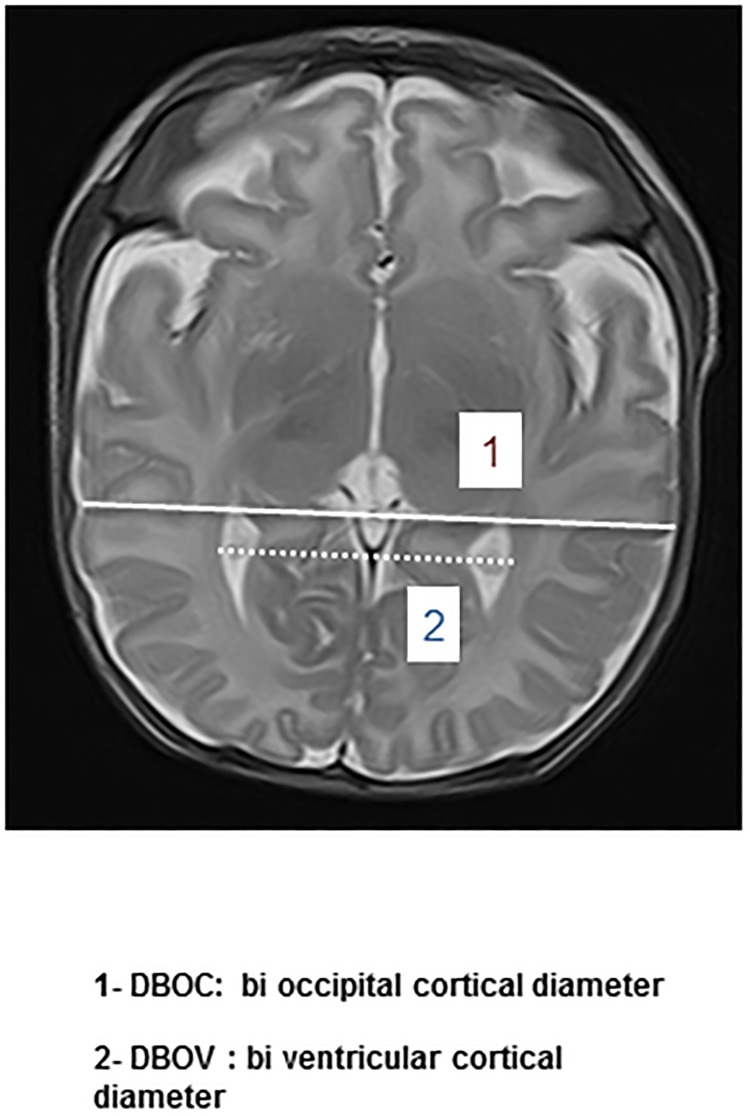
MRI linear indices on the axial image in T2-weighted including mesencephalon and occipital horn of lateral ventricle, below the thalamic nuclei. DBOv: bi occipital ventricular diameter / DBOc: bi occipital cortical diameter.

Secondly, additional measures were done on the axial image in T2-weighted including mesencephalon and occipital horn of lateral ventricular, below the thalamic nuclei. Two diameters were measured: the bi occipital ventricular diameter (DBOv) defined as the largest diameter at the occipital horns; and the bi occipital cortical diameter (DBOc) (Fig 2).

An arithmetic average (MA) was calculated at the cortex (MAc) and the skull (MAs) level by the formula: (DAP+DT)/2. An arithmetic average was also calculated for the thickness of lateral ventricle (MAfhlv) by the formula: ((FHLVL+FHLVR)/2).

The following linear indices were defined: cortex-skull index (CSI = MAc/MAs), ventricular-cortex index (VCI = DBVP/DTc), and bi occipital index (BOI = DBOv/DBOc). Finally, approximating the brain shape as a sphere, we calculated a volume slice area (VS.A) by the formula ((MAc)/2) ^2 x 3.14) as an indicator of the cerebral volume. All measures were realized by two independent investigators (an experienced neuroradiologist and a pediatric resident) in a blinded manner.

MRI were categorized as “MRI globally normal” or “MRI globally abnormal” when there were lesions such as supra-tentorial injury (sequelae of germinal matrix hemorrhage, sub ependymal hemorrhage and diffuse or cystic periventricular leukomalacia), sub tentorial or fossa posterior lesions (cerebellar hemorrhagic micro bleeds) as described by Inder et al. [6,7].

### Clinical and pharmacological data

Data related to the neonatal period were collected from the medical file of the infants in a standardized manner. The characteristics of the infants (gestational age, gender, Apgar score, anthropometrics parameters), the therapeutics used (corticosteroids, vasopressive drugs, cardio-respiratory treatment, diuretics, drugs acting on the central nervous system (midazolam, sufentanil), non-steroidal anti-inflammatory (NSAI), antibiotics, red blood cells and platelet transfusions), and the major complications of the prematurity (patent ductus arteriosus (PDA), IVH (diagnosed by intracranial ultrasound and defined after Papiles’ classification), c-PVL (diagnosed by MRI), bronchopulmonary dysplasia defined as the need for oxygen therapy at 28 days postnatal age (BPD), retinopathy of prematurity (ROP) according to international classification, necrotizing enterocolitis defined by Bell, early and late onset infections) were recorded.

### Statistical analyses

Inter-observer differences were estimated by calculating the mean and 95% confidence interval (CI) of the arithmetic differences between measurements on the same subject. Magnitude of the differences was evaluated by the ratio between the absolute value of the mean arithmetic of the differences and the mean value of the measured parameter. Variability was calculated as 1.96 standard deviation (SD) of the absolute value of the mean arithmetic of the differences according to Bland and Altman [20, 21], as described by Singh et al [22]. For subsequent analysis, the average of the 2 measures was taken into account.

Normally distributed data, assessed by a Shapiro-Wilk test of normality, are presented as mean values with SD, the median and the interquartile range (IQR); non-normally distributed data are presented as median with IQR only. To evaluate differences between groups, we used the Student’s *t* test for continuous variables and the Chi2-test (or Fisher exact test when appropriate), for categorical variables. For continuous variables not normally distributed, we used the Mann-Whitney U test. Significance level was set at 0.05.

As a first step, reference values were defined as the IQR of the data recorded from the MRI quoted “globally normal” by a neuroradiologist blinded for the measurements and the perinatal evolution of the infants.

As a second step, these values were compared with those of the MRI quoted “globally abnormal”, by bivariate analysis. Significance level was set at 0.05. Finally, confounding factors for each MRI index with a p-value <0.1 in bivariate analysis were included in a stepwise multiple linear regression model with a Tolerance set at 10^−5^, the probability to remove set at 0.15 and a Confidence Interval at 0.95. Statistical analysis was performed with SYSTAT 12 software (2007, Systat Software Inc. San Jose CA, USA).

## Results

### Population characteristics and MRI interpretation

Sixty nine infants were enrolled in this cohort study; one case was excluded for chromosome abnormality. Median (IQR) gestational age was 30.0 weeks gestation (27.0; 30.0); median weight birth was 1240 grams (986; 1477). There was no gender difference (36 boys/ 33 girls); 57% of the preterm infants received a complete course of antenatal corticotherapy. The median age for the realization of the MRI at TEA was 41.0 weeks (40.0; 42.0); 14 preterm infants had presented with IVH, and 14 with ROP, 5 infants having both IVH and ROP. Two premature infants had neonatal ventricular dilatation, defined as a measure of the frontal horn of the lateral ventricle over 5 mm. Sixty one premature infants (88%) required mechanical ventilation, 43.5% (n = 30) needed oxygen therapy at 28 days postnatal age. Eight infants required surgery for PDA; 29% presented with late onset sepsis.

Forty eight MRI (69.5%) were quoted normal or with punctual insignificant abnormality and 21 MRI were quoted abnormal (14 with supra-tentorial area abnormalities, 6 with supra-tentorial and posterior fossa injury, 1 preterm infant only presented with posterior fossa abnormalities). The characteristics of the two groups (MRI normal vs. abnormal) are presented in Table 1. Infants with abnormal MRI were significantly younger than premature infants with normal MRI. We observed a strong association between MRI abnormalities at TEA and IVH in the neonatal period (OR [0.95 CI] = 10.000 [2.633–37.978]; p = 0.001). There was also an association with ROP (OR = 4.205 [1.231–14.368]; p = 0.017), surgery for PDA (OR = 4.688 [1.01–21.89]; p = 0.036), early onset infection (OR = 4.688 [1.004–21.889]; p = 0.036) and neonatal treatment by cefotaxime (OR = 3.222 [1.093–9.497]; p = 0.03). The following factors were not significantly associated with MRI results: gender, mechanical ventilation, bronchopulmonary dysplasia, necrotizing enterocolitis, late onset sepsis, use the other treatments studied (corticosteroids, NSAI, diuretics, drugs acting on the central nervous system, red blood cells transfusion).

**Table 1:** Characteristics of premature infants with normal or abnormal MRI.

Median (IQR or %)	Normal MRI (48)	Abnormal MRI (21)	p
Gestational age	30.0 (29.0; 31.0)	28.0 (26.8; 30.0)	**0.002**
Birth weight	1250 (1002; 1458)	1160 (971; 1486)	0.870
Birth height	38.0 (37.0; 40.0)	38.0 (36.0; 40.3)	0.990
Birth head circumference	27.0 (25.3; 28.0)	26.5 (24.9; 27.3)	0.337
Apgar score at 1 min	5.0 (4.0; 7.0)	4.5 (2.5; 6.0)	0.198
Apgar score at 10 min	7.0 (6.0; 8.0)	7.0 (5.5; 7.5)	0.458
Age at MRI	41.0 (40.0; 42.0)	41.0 (40.0; 42.0)	0.815
Weight at MRI	3335 (2955; 3820)	3480 (2990; 3858)	0.611
Height at MRI	48.0 (46.0; 50.0)	49.0 (47.3; 50.0)	0.315
Head circumference at MRI	36.0 (34.5; 37.0)	36.0 (35.6; 36.9)	0.813
Antenatal corticosteroids	28 (58%)	11 (52%)	0.159
Postnatal corticosteroids	8 (17%)	6 (29%)	0.276
Mechanical ventilation	42 (87%)	19 (90%)	0.722
O2 need at 28 days PNA	18 (37%)	12 (57%)	0.130
IVH	4 (8%)	10 (48%)	**0.001**
Vasopressive drugs	4 (8%)	3 (14%)	0.451
NSAI (ductus arteriosus)	8 (17%)	7 (33%)	0.122
Surgery for PDA	3 (6%)	5 (24%)	**0.036**
Early onset infection	16 (33%)	5 (24%)	**0.036**
Late onset infection	13 (27%)	10 (48%)	0.096
Retinopathy of prematurity	6 (12%)	8 (38%)	**0.017**
Necrotizing enterocolitis	7 (15%)	1 (5%)	0.231

MRI: magnetic resonance imaging/ O2: oxygen/ PNA: postnatal age / IVH: intra ventricular hemorrhage/ NSAI: nonsteroidal anti-inflammatory/ PDA: patent ductus arteriosus

### Measures and reproducibility of the measures

No statistical difference was noted between the measures of the two independent investigators: the most important variability was observed for DBOc, measured at 2.3 mm. The comparison between the measures of 2 investigators is presented in Table 2.

**Table 2:** Differences between measures of two investigators.

	Arithmetic mean (mm)	Confidence limit at 95.0%	Magnitude of the differences	Variability (mm)
**DAPc**	0.793	0.586; 1.000	0.007	1.69
**DTc**	0.210	0.022; 0.398	0.002	1.53
**DAPs**	-0.064	-0.229; 0.102	0.001	1.34
**DTs**	-0.193	-0.334; -0.052	0.002	1.15
**FHLVR**	0.187	0.077; 0.297	0.087	0.896
**FHLVL**	0.249	0.121; 0.378	0.084	0.896
**DBVP**	-0.391	-0.667; -0.115	0.011	2.250
**DBOv**	0.377	0.113; 0.640	0.009	2.150
**DBOc**	1.097	0.812; 1.382	0.013	2.328

DAPc: antero posterior diameter of cortex /DAPs: antero posterior diameter of skull/ DTc: transversal diameter of cortex / DTs: transversal diameter of skull/ FHLVR: thickness of frontal horn of right lateral ventricle/ FHLVL: thickness of frontal horn of left lateral ventricle/ DBVP: biventricular posterior diameter/ DBOv: bi occipital ventricular diameter / DBOc: bi occipital cortical diameter.

The reproducibility being acceptable the average of the measures of both investigators was used for the rest of the study. Mean values of the measures for the 69 infants, median and IQR, representing the reference values for this population, are presented in Table 3.

**Table 3:** Average of raw measures.

	Arithmetic mean	Median	Inter Quartile Range
**DAPc**	107.5	107.7	104.5; 110.5
**DTc**	84.1	83.5	81.4; 87.5
**DAPs**	111.3	110.5	108.2; 114.6
**DTs**	86.6	85.7	84.0; 89.8
**FHLVR**	2.2	2.0	1.2; 3.0
**FHLVL**	2.9	2.7	1.5; 3.8
**DBVP**	34.9	34.9	33.0; 36.6
**DBOv**	43.4	43.1	41.7; 45.3
**DBOc**	82.1	81.8	79.3; 85.2

Dapc: antero posterior diameter of cortex /DAPs: antero posterior diameter of skull/ DTc: transversal diameter of cortex / DTs: transversal diameter of skull/ FHLVR: thickness of frontal horn of right lateral ventricle/ FHLVL: thickness of frontal horn of left lateral ventricle/ DBVP: biventricular posterior diameter/ DBOv: bi occipital ventricular diameter / DBOc: bi occipital cortical diameter.

There was no statistical difference of these raw measures between normal and globally abnormal MRI at supra-tentorial level. Conversely, for lesions of the posterior fossa (PF), we observed a significant increase in two measures of the occipital area (DBVP and DBOv) (Table 4). Infants with ROP had four measures significantly shorter (DTc, DTs, DBOv, DBOc) than healthy preterm infants. All these measures are representative of the posterior area.

**Table 4:** Measures with or without posterior fossa lesion and retinopathy.

*PF LESIONS*			
	**Posterior fossa lesions (n = 7)**	**Normal posterior fossa (n = 62)**	**p**
**DBVP**	36.4 (34.9; 40.2)	34.7 (32.6; 36.6)	0.032
**DBOv**	45.4 (43.2; 48.6)	43.2 (40.9; 42.2)	0.046
*ROP*			
	**Retinopathy (n = 14)**	**No retinopathy (n = 54)**	**p**
**DTc**	81.5 (78.7; 83.6)	84.7 (81.9; 88.1)	0.016
**DTs**	83.7 (81.1; 85.5)	87.3 (84.8; 90.3)	0.007
**DBOv**	42.2 (40.9; 43.2)	43.7 (42.3; 45.6)	0.049
**DBOc**	79.1 (76.8; 80.9)	82.9 (80.0; 85.8)	0.009

PF: posterior fossa/ ROP: retinopathy of prematurity/ DTc: transversal diameter of cortex / DTs: transversal diameter of skull / DBVP: biventricular posterior diameter/ DBOv: bi occipital ventricular diameter / DBOc: bi occipital cortical diameter.

### Cerebral indices and determinants

Five different indices were calculated from the linear measures (CSI, MAfhlv, VCI, BOI and VS.A). The characteristics of these indices are presented in Table 5.

**Table 5:** Cerebral indices characteristics.

	Arithmetic mean	Median	IQR
**CSI**	0.968	0.969	0.963; 0.975
**MAfhlv (mm)**	2.537	2.375	1.450; 3.363
**VCI**	0.415	0.418	0.394; 0.434
**BOI**	0.529	0.531	0.515; 0.541
**VS.A (mm**^**2**^**)**	7216	7257	6930; 7553

IQR: interquartile range; CSI: cortex-skull index/ MAfhlv: arithmetic average of the thickness of lateral ventricle/ VCI: ventricular-cortex index/ BOI: bi occipital index/ VS.A: volume slice area

There was a statistical difference for **VCI** between normal and abnormal MRI (0.412 (0.388; 0.429) vs. 0.432 (0.418; 0.449); p = 0,019). This difference persisted in premature infants with supra-tentorial lesions (0.414 (0.390; 0.430) vs. 0.432 (0.418; 0.450); p = 0.031) or fossa posterior abnormality (0.417 (0.391; 0.431) vs. 0.443 (0.436; 0.450); p = 0,026). Also, for the fossa posterior, **BOI** was significantly higher in case of abnormalities (0.530 (0.513; 0.540) vs. 0.543 (0.534; 0.553); p = 0.022). There was no statistically difference of these indices for IVH, ventricular dilatation and preterm infants with a retinopathy.

Tables 6 and 7 summarize the bivariate analysis and the models obtained by stepwise multiple linear regression of significant variables for cerebral indices.

**Table 6:** Bivariate analysis significant determinants of cerebral indices.

P values	CSI	MAfhlv	VCI	BOI	VS.A
**Gender**		0.047			
**Gestational Age**				0.027	0.001
**Birth Weight**					0.001
**Birth Head circumference**					0.001
**Postmenstrual age**		0.014			0.001
**TEA Weight**		0.013			0.001
**TEA Head Circumference**		0.001			0.001
**Bronchopulmonary dysplasia**					0.015
**NSAI (ibuprofen)**					0.002
**Surgery ductus arteriosus**			0.038	0.04	0.015
**Late onset sepsis**				0.033	0.048
**Toxics**		0.03			0.027
**Tobacco**		0.03			0.013
**Antenatal corticoids**				0.03	
**Postnatal corticoids**					0.001
**Dopamine**		0.189	0.057	0.027	0.068
**Midazolam**					0.013
**Furosemide**	0.025				0.004
**Amoxicillin**		0.005			
**Vancomycin**				0.021	0.002
**Doxapram**			0.001	0.034	
**Blood transfusion**			0.197	0.051	0.06
**Platelets transfusion**					0.027
**Ventricular dilatation**		0.041	0.053	0.035	
**Hyperbilirubinemia**	0.108	0.07	0.023		
**Apgar score <3 at 1min**				0.072	

CSI: cortex-skull index/ MAfhlv: arithmetic average of the thickness of lateral ventricle/ VCI: ventricular-cortex index/ BOI: bi occipital index/ VS.A: volume slice area/ TEA: term equivalent age/NSAI: non-steroidal anti-inflammatory

**Table 7:** Summarizes the stepwise multivariate analysis of determinants of all cerebral indices.

Cerebral Index (Multiple r)	Determinants	Effect (coefficient)	0.95 C.I.	P value
CSI **(r = 0.271, p = 0.025)**	Furosemide	0.008	0.001;0.015	0.025
MAfhlv **(r = 0.616, p<0.001)**	Gender	0.734	0.248;1.220	0.004
	TEA head circumference	0.260	0.073;0.447	0.007
	Amoxicillin	- 0.559	- 1.098;-0.020	0.042
	Late onset sepsis	- 0.505	- 1.088;0.078	0.088
	Ventricular dilatation	- 1.274	- 2.768;0.220	0.093
VCI **(r = 0.462, p<0.001)**	Hyperbilirubinemia	0.022	0.004;0.040	0.018
	Doxapram	- 0.022	- 0.036;-0.009	0.001
BOI **(0.465, p = 0.002)**	Ventricular dilatation	- 0.044	- 0.073;-0.015	0.014
	Postmenstrual age	- 0.003	- 0.006;0.000	0.061
	Apgar score <3 at 1min	- 0.009	- 0.020;0.003	0.127
VS.A **(r = 0.894; p<0.001)**	TEA head circumference	262.9	200.9;324.9	<0.001
	Postmenstrual age	89.1	34.7;143.5	0.002
	Dopamine treatment	375.2	140.2;610.2	0.002
	Blood transfusion	- 303.2	- 501.7;-104.7	0.003
	Ibuprofen treatment	231.1	15.7;446.6	0.036
	Birth head circumference	51.2	- 1.1;103.5	0.055
	Platelets transfusion	211.5	- 57.5;480.4	0.121

VS.A appears to be the best surrogate for cerebral volume (approximating the brain shape as a sphere); 80% of VS.A are explained by a model including seven linked variables (r = 0.894; p<0.001). Two of them are anatomic (head circumference at birth and at TEA), one also influenced MAfhlv (head circumference at TEA); one variable reflects preterm infant maturity (post menstrual age), and four variables are treatments of prematurity complications (dopamine, ibuprofen for patent ductus arteriosus treatment, blood and platelets transfusions).

Of note, other therapeutics studied were determinants for linear indices: furosemide for CSI, amoxicillin for MAfhlv, doxapram for VCI. Ventricular dilatation was associated with BOI. Gender was only determinant for MAfhlv. Ante or postnatal steroids were not determinants for our indices, in stepwise multiple linear regression.

## Discussion

In this study, we defined five cerebral indices from simple and reproducible linear measures. The good inter-investigator reproducibility of these indices is consistent with measures easily and rapidly achievable. These indices might be very useful when evaluating specific situations. For instance, Ragan et al. showed that the ventricular volume in infants presenting with hydrocephaly had a strong correlation with a linear index easier to measure than cerebral volume itself: FOHR, which adds the two ventricular diameter of frontal and occipital horns of the lateral ventricles (equivalent to our index, DBVP), divided by twice the transversal diameter of cortex (equivalent to our index, DTc) [19]. Pineda et al studied brain structure differences in neonatal intensive care between open ward and private room in preterm infants with qualitative brain measures, brain metrics (bifrontal, biparietal, transcerebellar diameters, ventricular size and interhemispheric distance), diffusion measures, and volumetry [23]. These methods require trained personnel, specific equipment and cannot be applied in clinical routine. Kidokoro et al. [24] and Inder et al. [25] used a standardized scoring system to evaluate cerebral white matter and cortical gray matter abnormalities. Our study is a quantitative global MRI analysis. We objectively measured parenchyma and skull length and created indices to reflect changes in brain volume independent of changes in brain shape. We studied five indices: four linear indices representing different brain regions and a surface index. All these data are easily obtained when performing standard MRI at TEA.

Thus, our study proposes interquartile ranges for measures and indices. These values need to be validated in a large population as they would allow generalization for diagnosis and follow-up of former premature infants with risk factors of brain anomalies.

In our population, the surface index seems to be the best surrogate for cerebral volume. VS.A is explained, for 80% of its variance, by a model including seven well defined linked variables. Head circumference at TEA is an independent factor for VS.A which is consistent with anatomic observation and previous publications. Head circumference measures are routinely used for evaluating cerebral growth. Treit et al. demonstrated on children with prenatal alcohol exposure that head circumference was positively correlated with brain volume [26]. Bartholomeusz et al. showed an age dependent relationship between head circumference and cerebral volume [27], the head circumference growth being positively associated with brain volume.

Some therapeutics used during the neonatal period are also determinant for the model explaining VS.A: dopamine, ibuprofen and blood transfusion. Loeliger et al. showed that the treatment by ibuprofen was not associated with an alteration of brain growth but this study was realized in the preterm baboon [28]. Surprisingly, corticosteroid use was not a determinant of VS.A in our population whereas several studies have shown a relationship of this treatment with brain volume [11, 15–17]. The lack of significance for corticosteroids, by multivariate analysis in our study, may be related to the size of our cohort which was not powered to answer that question. In addition, it is difficult to separate a treatment from the reason it was used and different policies of treatment may lead to different results. Nowadays, the prevalence of postnatal corticosteroids use has significantly decreased, as compared to the years before 2005 when most of the publications were written, which may also explain this difference.

Ventricular dilatation was a significant determinant of BOI, this index measuring the lateral ventricles.

VCI values were statistically different between normal MRI and abnormal MRI. This difference persists for supra-tentorial and posterior fossa lesions. At supra-tentorial level, periventricular leukomalacia, the second more frequent cerebral complication of prematurity, after intra ventricular hemorrhage, is responsible for white matter atrophy consequence of passive ventricular dilatation. It is made of ischemic lesions of white matter adjacent to the external edges of the lateral ventricles related to apoptosis and necrosis [29] but also to disorders in the development and neuronal differentiation [30]. Inder et al. demonstrated that premature infants with leukomalacia had a reduction in cerebral cortical gray matter at TEA, compared with either premature without leukomalacia or with normal term infants (157.5 ml with c-PVL versus 211.7 ml without c-PVL versus 218.8 ml normal term infants) [7]. For posterior fossa injury, BOI was also significantly different. We did not observe any significant difference with regards to indices on infants presenting with retinopathy of prematurity.

In our population, abnormal MRI was related to surgery for PDA failing ibuprofen use, ROP and early onset infection (and, logically, with cefotaxime treatment). These results are consistent with the literature. Galli et al. demonstrated that periventricular leukomalacia was found in 54% of neonates after cardiac surgery (for congenital heart defects) with continuous cardiopulmonary bypass in relation to hypoxemia and hypotension [31]. Parikh et al. found, for extremely low birth weight infants, that surgery for retinopathy of prematurity was associated with an increased volume of white matter hyper intensities at TEA [32]. Rand et al. showed an association between confirmed neonatal infection of very preterm infants and neurodevelopmental impairment via white matter abnormalities [33].

Our study has limitations. One of them is the relative small size of our cohort which can lead to a lack of power for variables with low prevalence. Studies with larger population are needed to validate our findings and allow generalization. Also, we could not compare VS.A to the gold standard, cerebral volume itself. Therefore, we cannot prove that VS.A is the best surrogate for cerebral volume. However, with regard to the reproducibility and the ease of establishing this surface index, this validation would be very interesting.

Nine MRI (13%) required neuroradiologist’s re-evaluation because there were difficult to classify as normal or globally abnormal.

De Vries et al. demonstrated a strong connection between MRI at TEA and the neurologic outcome of the infants [4]. Keunen et al showed that the cerebellar volume at TEA was positively correlated to cognitive development at 24 months and 3.5 years [34]. Monson et al observed that a small brain volume at TEA in preterm infants is exaggerated at 7 years. Also, there was an association between low brain volume in infancy and long-term functional outcome [35]. Thus, it would be interesting to study the evolution at short and long term of our infants, to evaluate the prognosis value of these indices with their neurodevelopment outcome.

## Conclusion

The screening of children at risk of presenting with neurodevelopmental disorders is important for accurately answering parents’ concern and for selection of infants who may benefit from early intervention. Our study defined indices, easily and rapidly achievable in routine MRI, which may improve MRI specificity. The surface index seems to be a good surrogate for cerebral volume. This study need to be validated in a large population to allow generalization for the follow-up of former premature infants.
